# Genomic Epidemiology of Large Blastomycosis Outbreak, Ontario, Canada, 2021

**DOI:** 10.3201/eid3007.231594

**Published:** 2024-07

**Authors:** Lisa R. McTaggart, Nobish Varghese, Karthikeyan Sivaraman, Samir N. Patel, Julianne V. Kus

**Affiliations:** Ontario Agency of Health Protection and Promotion (Public Health Ontario), Toronto, Ontario, Canada (L.R. McTaggart, N. Varghese, K. Sivaraman, S.N. Patel, J.V. Kus);; University of Toronto, Toronto (S.N. Patel, J.V. Kus)

**Keywords:** blastomycosis, genomic epidemiology, outbreak, *Blastomyces gilchristii*, MycoSNP, dimorphic fungi, fungi, respiratory infections, Ontario, Canada

## Abstract

Using phylogenomic analysis, we provide genomic epidemiology analysis of a large blastomycosis outbreak in Ontario, Canada, caused by *Blastomyces gilchristii*. The outbreak occurred in a locale where blastomycosis is rarely diagnosed, signaling a possible shift in geographically associated incidence patterns. Results elucidated fungal population genetic structure, enhancing understanding of the outbreak.

North American blastomycosis is an infection most commonly caused by environmental dimorphic fungi *Blastomyces dermatitidis* and *B. gilchristii*. Infections range from asymptomatic to severe, typically presenting as respiratory illness, with possible systemic dissemination (*1*,*2*). The geographic range of *B. dermatitidis* and *B. gilchristii* fungi spans the eastern half of North America, including Ontario, Canada (*2*,*3*). Although overall incidence rates are low, isolated cases of blastomycosis are diagnosed regularly among populations in endemic areas (*2*,*3*), and clusters and outbreaks occur due to common environmental exposures (*1*,*4*–*7*).

We describe a large genomic epidemiology investigation of a blastomycosis outbreak in Constance Lake First Nation, a small community (population <2,000) in northeastern Ontario, Canada, in a locale where blastomycosis has rarely been encountered (*2*). We studied samples from 181 patients that were received by the Public Health Ontario Laboratory during November 2021–May 2022. By August 2022, we identified *B. gilchristii* fungus, by using multilocus sequence typing (*8*), in cultures from 40 persons linked to the outbreak (37 community residents and 3 persons [deemed travel A, B, and C] who visited the community during the possible exposure window). We observed that most positive cultures (35/40) were derived from specimens collected during a 7-week period—mid-November 2021 through December 2021—and most (39/40) were obtained from respiratory specimens (Table). Patients spanned all age groups; 55% were male and 45% female (Table).

**Table:** Clinical characteristics of culture-confirmed outbreak cases from a large blastomycosis outbreak, Ontario, Canada, 2021

Characteristic	Culture-confirmed cases, no.
Patient age, y	
0–19	13
20–34	7
35–49	13
50–69	7
Patient sex	
M	22
F	18
Date collected	
2021 Nov	23
2021 Dec	12
2022 Jan–Aug	5
Specimen type	
Sputum	37
Other	3

We performed whole genome short-read sequencing (Illumina, https://www.illumina.com) on outbreak isolates and 21 other randomly selected *B. gilchristii* isolates (Appendix) cultured from specimens of patients from northern and eastern Ontario in July 2019–July 2022 (BioProject no. PRJNA890593). This collection of sequenced isolates contributes substantially to the number of *B. gilchristii* genomes available to advance research on this important pathogen. We conducted single-nucleotide variant (SNV) analysis to determine genetic diversity and relatedness between the 61 Ontario isolates plus 4 isolates from patients in Minnesota, USA (BioProject no. PRJNA786864), by using MycoSNP v0.22 (https://github.com/CDCgov/mycosnp-nf) with reference genome *B. gilchristii* SLH14081 (GenBank accession no. GCA_000003855.2) and conducted phylogenomic analysis (neighbor-joining method) using MEGA11 (https://www.megasoftware.net) (*1*).

Phylogenomic analysis of SNVs suggested that 39 of the 40 outbreak isolates were genetically highly similar, including isolates from 2 nonresidents who had traveled to the impacted community (travel A and B) (Figure). By contrast, the isolate from travel C was genetically dissimilar from the other outbreak isolates and likely represents a sporadic case acquired elsewhere (Figure). High genetic similarity between outbreak isolates and the brief 7-week timeframe for symptom onset for 90% of cases suggests a shared discrete exposure window. The overall analysis depicts multiple genetically distinct populations of *B. gilchristii* fungi separated by thousands of SNVs, correlated with different geographic regions.

**Figure:**
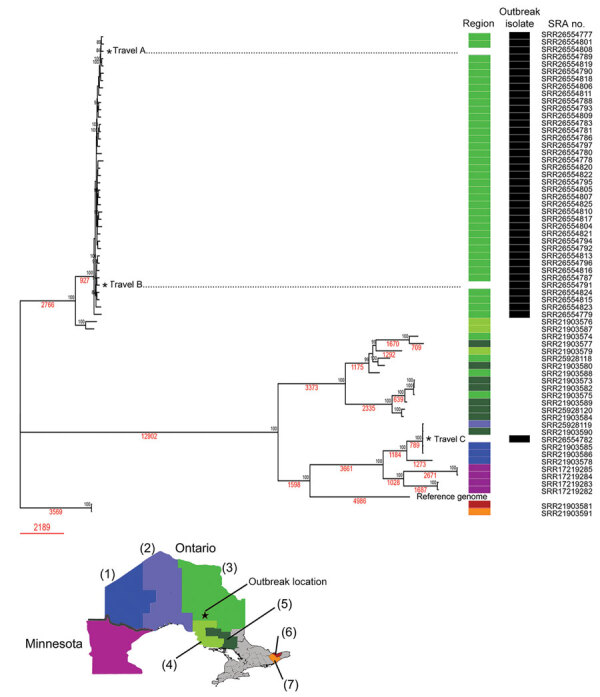
Phylogenomic analysis of whole-genome single-nucleotide variants by neighbor-joining method of *Blastomyces gilchristii* isolates from a large blastomycosis outbreak, Ontario, Canada, 2021. Percentage of trees of 500 bootstrap replications in which the associated taxa clustered together is shown next to the nodes. The tree is drawn to scale, with branch lengths measured in number of substitutions per site (red text). There were 45,321 positions in the final dataset. Outbreak isolates are designated with black bars. Colors indicate geographic region in which the patient resided is as shown on map, including cases from Minnesota, USA; numbers indicate regions: (1) Northwest, (2) Thunder Bay District, (3) Porcupine, (4) Algoma, (5) Sudbury, (6) Ottawa, (7) Leeds/Grenville/Lanark. The geographic regions of residence for the travel cases were not available. SRA, National Center for Biotechnology Information Sequence Read Archive.

To validate the number of SNV differences between isolates, we separately investigated 9 pairs of biologic replicates (isolates from separate specimens from the same person) and 3 technical replicates from 2 other randomly selected isolates (Appendix). We found that 8 of 9 biologic replicates and all technical replicates averaged 12 (range 5–20) SNVs between corresponding isolates. In contrast, the number of SNVs between 39 outbreak isolates (excluding travel C) averaged 380 (range 96–778); 1 pair of biologic replicates differed by 192 SNVs. By comparison, 5 other clusters of isolates, including 2 clusters from Minnesota patients, had <100 SNVs between isolates. Taken together with the timing of the outbreak, the SNV differences between isolates suggests a shared exposure to a genetically similar yet heterogeneous fungal population, rather than a genetically identical point source. We theorize that the pair of biologic replicates differing by 192 SNVs might represent infection by 2 different strains of the fungal population. Of note, the outbreak locale possesses several environmental niches considered suitable reservoirs for *Blastomyces* spp. fungi, namely waterway-adjacent and forested areas, with coniferous trees supplying acidic soil rich in decaying organic material (*7*).

During the past 40 years, reports have documented several outbreaks and case clusters of North American blastomycosis (*1*,*4*–*7*). Most ascribe exposure to a discrete time and environmental locale, although that presumption is challenging to confirm because of the variable incubation time (1–6 months) (*6*) and patient travel. Recently, genomic epidemiologic information gleaned from whole-genome bioinformatics analysis has enhanced the description of outbreaks and case clusters of fungal pathogens (*9*,*10*), including *B. gilchristii* (*1*), enabling a more fulsome understanding of disease acquisition. In this study, we have interpreted the genomic epidemiology of a large outbreak of *B. gilchristii* within the context of several contemporaneous, genetically unrelated isolates to describe pathogen population genetic structure. We consider such genomic epidemiologic analyses useful in identifying cases caused by genetically related strains and, when combined with other epidemiologic factors including patient travel, pinpointing the timing and location of exposure. Epidemiologic investigation combined with pathogen phylogenomic analyses enables improved understanding of blastomycosis outbreaks and disease dynamics. This detailed information might help elucidate ecologic variables (possibly altered by climate change or modified land-use patterns) that influence disease acquisition (*3*). We believe that increased awareness of pathogen range and risk can aid prompt future case diagnoses.

Appendix Additional information on genomic epidemiology of large blastomycosis outbreak, Ontario, Canada, 2021.
