# Supplementary material for: Genomic Epidemiology of Large Blastomycosis Outbreak, Ontario, Canada, 2021
Source: Emerg Infect Dis. 2024 Jul;30(7):1487–90. doi: 10.3201/eid3007.231594 (PMC11210661; doi:10.3201/eid3007.231594)
Supplement: Appendix — Additional information on genomic epidemiology of large blastomycosis outbreak, Ontario, Canada, 2021. [file 23-1594-Techapp-s1.pdf]

Article DOI: http://doi.org/10.3201/eid3007.231594

EID cannot ensure accessibility for supplementary materials supplied by authors. Readers who have difficulty accessing supplementary content should contact the authors for assistance.

Genomic Epidemiology of Large Blastomycosis Outbreak, Ontario, Canada, 2021

Appendix

Appendix Table. Description of study isolates and BioProject PRJNA890593 sequence read accession (SRA) numbers from an analysis of genomic epidemiology of large blastomycosis outbreak, Ontario, Canada, 2021*

Sample no. (Travel case)	SRA no.	Outbreak isolate	Duplicate†	Collection date	Specimen type	Geographic region (Ontario Public Health unit)
21X008	SRR26554818	yes	na	2021-Nov	sputum	Porcupine
21X012	SRR26554788	yes	na	2021-Dec	sputum	Porcupine
21X030	SRR26554824	yes	na	2021-Nov	sputum	Porcupine
21X039 (Travel A)	SRR26554808	yes	na	2021-Nov	sputum	not available
21X054	SRR26554813	yes	na	2021-Nov	sputum	Porcupine
21X057	SRR26554792	yes	na	2021-Nov	sputum	Porcupine
21X063	SRR26554817	yes	na	2021-Nov	sputum	Porcupine
21X118	SRR26554816	yes	na	2021-Nov	sputum	Porcupine
21X121	SRR26554815	yes	na	2021-Nov	sputum	Porcupine
21X123	SRR26554811	yes	na	2021-Nov	sputum	Porcupine
21X315	SRR26554786	yes	na	2021-Dec	sputum	Porcupine
21X460	SRR26554821	yes	na	2021-Dec	respiratory specimen	Porcupine
21X587	SRR26554806	yes	na	2021-Dec	respiratory specimen	Porcupine
21X590	SRR26554787	yes	na	2021-Dec	sputum	Porcupine
21X701 (Travel B)	SRR26554791	yes	na	2021-Dec	sputum	not available
21X758	SRR26554805	yes	na	2021-Dec	sputum	Porcupine
21X848	SRR26554807	yes	na	2021-Dec	sputum	Porcupine
21X866	SRR26554796	yes	na	2021-Nov	sputum	Porcupine
21X867	SRR26554781	yes	na	2021-Nov	sputum	Porcupine
21X869	SRR26554801	yes	na	2021-Nov	sputum	Porcupine
21X871	SRR26554795	yes	na	2021-Nov	sputum	Porcupine
21X883	SRR26554825	yes	na	2021-Nov	sputum	Porcupine
21X914	SRR26554793	yes	na	2021-Nov	sputum	Porcupine
21X923	SRR26554823	yes	na	2021-Nov	sputum	Porcupine
21X930	SRR26554820	yes	na	2021-Nov	sputum	Porcupine
22X011	SRR26554780	yes	na	2022	other	Porcupine
22X115 (Travel C)	SRR26554782	yes	na	2022	sputum	not available
22X253	SRR26554779	yes	na	2022	sputum	Porcupine
22X720	SRR26554778	yes	na	2022	sputum	Porcupine
21X565	SRR26554775	yes	Patient A[1]	2021-Nov	respiratory specimen	Porcupine
21X870	SRR26554797	yes	Patient A	2021-Nov	sputum	Porcupine
21X127	SRR26554809	yes	Patient B	2021-Nov	sputum	Porcupine
21X264	SRR26554776	yes	Patient B	2021-Dec	sputum	Porcupine
21X955	SRR26554798	yes	Patient C	2021-Dec	sputum	Porcupine
21X998	SRR26554819	yes	Patient C	2021-Nov	sputum	Porcupine
21X288	SRR26554794	yes	Patient D	2021-Dec	sputum	Porcupine
21X776	SRR26554803	yes	Patient D	2021-Dec	sputum	Porcupine
21X7289	SRR26554774	yes	Patient E	2021-Dec	sputum	Porcupine
21X933	SRR26554822	yes	Patient E	2021-Nov	sputum	Porcupine
21X258	SRR26554804	yes	Patient F	2021-Dec	sputum	Porcupine
21X760	SRR26554773	yes	Patient F	2021-Dec	sputum	Porcupine
21X917	SRR26554799	yes	Patient G	2021-Dec	sputum	Porcupine

Sample no. (Travel case)	SRA no.	Outbreak isolate	Duplicate†	Collection date	Specimen type	Geographic region (Ontario Public Health unit)
21X942	SRR26554789	yes	Patient G	2021-Dec	sputum	Porcupine
21X290	SRR26554790	yes	Patient H	2021-Dec	sputum	Porcupine
21X849	SRR26554800	yes	Patient H	2021-Dec	sputum	Porcupine
21X840	SRR26554802	yes	Patient I	2022	sputum	Porcupine
22X096	SRR26554777	yes	Patient I	2022	sputum	Porcupine
21X125	SRR26554810	yes	TechRep1	2021-Nov	sputum	Porcupine
21X125B	SRR26554814	yes	TechRep1	2021-Nov	sputum	Porcupine
21X125C	SRR26554812	yes	TechRep1	2021-Nov	sputum	Porcupine
21X350	SRR26554783	yes	TechRep2	2021-Dec	sputum	Porcupine
21X350B	SRR26554785	yes	TechRep2	2021-Dec	sputum	Porcupine
21X350C	SRR26554784	yes	TechRep2	2021-Dec	sputum	Porcupine
19X156	SRR21903574	no	na	2019-Mar	sputum	Porcupine
19X159	SRR21903581	no	na	2019-Jul	BAL	Ottawa
19X280	SRR21903589	no	na	2019-Jul	sputum	Sudbury
19X301	SRR21903590	no	na	2019-Nov	sputum	Sudbury
19X542	SRR21903588	no	na	2019-Jul	bronchial washing fluid	Porcupine
19X611	SRR21903591	no	na	2019-Jul	BAL	Leeds, Grenville, Lanark
20X036	SRR21903586	no	na	2020-Nov	sputum	Northwest
20X504	SRR21903587	no	na	2020-Aug	sputum	Algoma
20X548	SRR21903584	no	na	2020-Nov	sputum	Sudbury
20X814	SRR21903582	no	na	2020-Aug	BAL	Sudbury
20X822	SRR21903585	no	na	2020-Sep	sputum	Northwest
21X285	SRR21903577	no	na	2021-Oct	bronchial washing fluid	Sudbury
21X289	SRR21903573	no	na	2021-Aug	ankle aspirate	Sudbury
21X331	SRR21903580	no	na	2021-Oct	sputum	Sudbury
21X378	SRR21903576	no	na	2021-Nov	BAL	Algoma
21X597	SRR21903578	no	na	2021-Jul	sputum	Northwest
21X805	SRR21903575	no	na	2021-Jan	sputum	Porcupine
21X982	SRR21903579	no	na	2021-Jan	sputum	Algoma
22X325	SRR25928120	no	na	2022-Jul	BAL	Sudbury
22X343	SRR25928119	no	na	2022-Apr	bronchial washing fluid	Thunder Bay District
22X864	SRR25928118	no	na	2022-Apr	BAL	Porcupine

*Hosts in all instances were homo sapiens. BAL, bronchoalveolar lavage fluid; na, not applicable.
†Patient identifiers were randomly assigned
